# Mesenchymal Stem Cells as a Feeder Layer Can Prevent Apoptosis of Expanded Hematopoietic Stem Cells Derived from Cord Blood

**Published:** 2014

**Authors:** Roya Mehrasa, Hamidreza Vaziri, Arezoo Oodi, Mona Khorshidfar, Mahin Nikogoftar, Monireh Golpour, Naser Amirizadeh

**Affiliations:** 1 *University of Guilan, Rasht, Iran.*; 2 *Blood Transfusion Research center, High institute for Research and Education in Transfusion Medicine,Tehran, Iran.*; 3 *Cellular and Molecular Biology Research Center, Babol University of Medical Sciences, Babol, Iran*.

**Keywords:** Cord blood expansion, apoptosis, co-culture, mesenchymal stem cell

## Abstract

Umbilical cord blood (UCB) has been used for transplantation in the treatment of hematologic disorders as a source of hematopoietic stem cells (HSCs). Because of insufficient number of cord blood CD34^+^ cells, the expansion of these cells seems to be important for clinical application. Mesenchymal stromal cells (MSCs), playing an important role in HSCs maintenance, were used as feeder layer. Apoptosis and cell cycle distribution of expanded cells were analyzed in MSCs co-culture and cytokine conditions and results were compared.

Three culture conditions of cord blood HSCs were prepared *ex-vivo* for 14 days: cytokines (SCF, TPO and Flt3L) with MSCs feeder layer, cytokines without MSCs feeder layer and co-culture with MSCs without cytokines. Expansion was followed by measuring the total nucleated cells (TNCs), CD34^+^^‏^ cells and colony-forming unit (CFU) output. Flow cytometry analysis of stained cells by annexin V and propidium iodide was performed for detection of apoptosis rate and cell cycle distribution in expanded cells. Maximum cord blood CD34^+^ cells expansion was observed in day 10. The mean fold change of TNCs and CD34+ cells at day 10 in the co-culture system with cytokines was significantly higher than the cytokine culture without MSCs feeder layer and co-culture system without cytokines (n=6, p=0.023). The highest apoptosis rate and the least number of cells in Go/G1 phase were observed in cytokine culture without feeder layer (p=0.041). The expansion of cord blood HSCs on MSCs as a feeder layer resulted in higher proliferation and reduction in apoptosis rate.

Umbilical cord blood (UCB) has been used as a source of hematopoietic stem cells (HSCs) for complete or partial human leukocyte antigen-matched allogenic transplantation to treat hematologic diseases. Cryopreserved umbilical cord blood (UCB) has also been applied to reduce the risk of graft versus host disease (GVHD), treatment-related mortality (TRM) and improve survival. However, there are approximately 107 CD34+ cells in one unit of cord blood, whereas for a successful transplantation, 2.5×105 / kg CD34+ cells are required. Evidently, ex vivo expansion of UCB-HSCs is the most effective method to increase the number of CD34+ cells and improve the kinetics of UCB-HSCs engraftments (1-3). But HSCs differentiation to precursor cells during ex-vivo expansion leads to the reduction of primitive HSCs (4, 5). Life, death, self-renewal and differentiation of HSCs are the major procedures that regulate the numbers and lifespan of the HSCs pool which defect within these processes can contribute to haemopoietic insufficiencies and development of haemopoietic malignancies (6). In the normal hematopoiesis, the balance between cell loss (apoptosis and differentiation) and cell gain (proliferation and mitosis) can determine the HSC population size (7). Several factors have been identified as potential mechanisms associated with self-renewal and proliferation of HSC. Some external factors including GM-CSF, Epo, Flt3, Notch and tumor suppressor genes like p21 affect stem cell division and regulation of self-renewal (7). Apoptosis is a physiological process that controls tissue kinetics and homeostasis.

Apoptosis controls hematpoietic stem cells growth factors thereby preventing them from leukemo-genesis. Growth factors, cell-cell contact and intracellular genes expression are important factors that control apoptosis. Among the significant factors enrolled in apoptosis, proliferation and maintenance of HSCs in a primitive state, are growth factors and stromal cells in culture media (8). 

Physiologically, stromal layer cells produce soluble factors which maintain the primitive characteristics of HSCs and the direct interaction between the HSC and MSC can catalyze this process (9). In this study we evaluated the rate of HSCs proliferation and apoptosis in the presence of mesenchymal stromal cells, Flt3-L, SCF and TPO.

## Materials and Methods

**Isolation of CD34** ^+^ **Cells**

HSCs were collected from fresh human umbilical cord blood after obtaining written consent from normal full-term pregnant women, at the Iranian Blood Transfusion Organization and according to the ethics committee guidelines.

Due to the surplus of red blood cells in cord blood, the RBCs were precipitated using hydroxyethyl starch (Stem cell technologies-Ottawa, Canada ). Mononuclear cells (MNCs) were isolated by density gradient centrifugation on Ficoll-Hypaque (Bio sciences, Sweden). The MNCs were then incubated with antihuman CD34 conjugated with microbeads (Miltenyi Biotec, USA) for 30 min at 4°C. Then CD34+ cells were isolated by using a column in a magnetic field.

**Feeder Layer Culture**

Mesenchymal stromal cells (MSCs) were isolated from fresh bone marrow after obtaining informed consent from healthy donors (at Taleghani Hospital, Tehran, Iran). Briefly, MNCs were collected from 10 ml aspirated bone marrow by density gradient centrifugation on Ficoll-Hypaque (Bio sciences, Sweden) and were washed with MSCs culture medium (low glucose DMEM supplemented with penicillin and streptomycin). The MNCs were cultured in a 75 cm^2^ culture flask at 37°C. After 2–3 days, non-adherent cells were removed and adherent cells were left in MSCs culture medium until cells confluence achieved 80%. MSCs were characterized by flow cytometric analysis using monoclonal antibodies against CD105, CD44, CD166, CD90, CD34 and CD45 (Dako, Denmark). Potential Differentiation of MSCs was investigated by osteogenic differentiation kit (Chemicon, USA). As a cell feeder layer, MSCs were harvested with 0·25% trypsin-EDTA solution (Stem cell technology, Denmark), and 1×10^4^ cells were plated in 6-well plates. When cells reached more than 90% confluence in DMEM, they were washed with PBS and were placed in a serum free medium (Stem Span) to co-culture with HSCs.

**MSCs Osteogenic differentiation assay**

Osteogenic differentiation was performed on bone marrow derived MSCs. Thus, 5x10^4 ^cellswere seeded in 24-well plates coated with collagen/vitronectin. After 100% confluency DMEM enriched with FBS was replaced by medium containing 100 μM l- ascorbic acid-2-phosphate, 1M β-glycerophosphate and 1mM dexamethasone (Chemicon, USA). On day 14, the cells were fixed with 70% ethanol for 1 h at 4 °C and stained for 15 min with alizarin red-S (Sigma) at room temperature (RT). Alkaline phosphatase staining was performed with alkaline phosphatase kit (Sigma-Aldrich). Differentiated cells werefixed with acetone and fast blue RR salt in naphthol. AS-MX Alkaline and Mayer's Hematoxylin were used for staining and counterstaining respectively.

***Ex-vivo*** **expansion of CD34** ^+^ **enriched cells**

CD34^+^ enriched cells (1×10^5^) were cultured in 6 well plates (Nunc, Denmark) for 14 days in serum-free medium (Stem cell technology, Denmark) at 37°C under 5% of CO2 humidified air in three culture conditions : cytokines culture supplemented with SCF (50 ng/mL), TPO (50 ng/mL) and FLT3L (40 ng/mL) (Stem cell technology, Denmark), co-culture with MSCs feeder layer and above mentioned cytokines and co-culture with MSCs without any cytokines.

**Colony-forming cell assays**

Fresh CD34^+^cells and expanded cells at 10^th^ day of culture (1-2×10^3^) were seeded in semisolid culture (Metho Cult GF H4434, Stem Cell Technology) following the manufacturer's instruction. Cells were mixed with Methylcellulose-based media and purred in 35-mm Petri dishes and incubated at 37°C, 5% CO2 in a humidified incubator. After the 14^th^ day of culture, the number of colony was counted under the inverted microscope.

**Proliferative and phenotypic analysis**

Cells viability were determined at 0^th^, 5^th^, 10^th^, and 14^th^ day of culture by counting of hematopoietic cells in each well using trypan blue stain (Stem Cell Technologies, USA) and stem cell and lineage markers were analyzed by flow  cytometry (Partec, Germany) using monoclonal antibodies against CD2, CD19 (Dako, Denmark) to evaluate lymphoid lineage differentiation; CD14, CD15, and CD13 to evaluate myeloid differen-tiation; Glycophorin A to evaluate erythroid differentiation and CD34, CD38 to assess the percentage of stem cells. Flow cytometric analysis was performed by incubating harvested cells with different fluorescent conjugated monoclonal antibodies at 4°C for 30 minutes. Then the cells were washed in PBS and fixed with 2% paraformaldehyde (Sigma). Isotype controls were used in every experiment.

**Cell Cycle Distribution Analysis by Flow Cytometry**

Cell cycle distribution was evaluated at 10^th^ day of culture by flow cytometry. Prior to staining, 200 μl of 1×10^6 ^cells/ml were washed by phosphate buffered saline (PBS) and re-suspended in 200 μl of PBS. Cells were treated with 50 μl of RNase (1 mg/ml) and 100 μl propidium iodide (PI, 400 μg/ml) (Sigma-Aldrich, Spain) for 30 min at 37 °C in the darkness. The fluorescence of stained cells was analyzed by flow cytometry and relative gated cells in each cell cycle phase were determined. Data acquisition and analysis were performed using flowmacs 2.0 software.

**Apoptosis Analysis by Annexin V**

Apoptosis rate were evaluated at 10^th^ day of culture by Apoptosis kit (Bioscience, USA). 1×10^6 ^cells were washed by phosphate buffered saline (PBS) and re-suspended in 1x binding buffer. The cells were treated with 5 μl of fluorochrome conjugated Annexin V for 15 min at room temperature. These cells were washed and re-suspended in 1x binding buffer and then 50μl of propidium iodide solution was added. The fluorescence of stained cells was analyzed by flow cytometry after 4 hours.

**Statistical Analysis**

Results obtained from multiple experiments are expressed as the mean ± standard deviation (SD). The data were analyzed using the t-test. Probability values<0.05 defined significant differences between test points.

## Results

**MSCs Immunophenotyping**

Isolated bone marrow MSCs were characterized by flow cytometric analysis of specific surface antigens. MSCs were found to be positive for the following adhesion molecules: CD44, CD166 and CD105, CD90 which together were considered as markers for MSCs. The MSCs were negative for haematopoietic lineage markers, CD34 and CD45.

**Osteogenic differentiation assay**

**Fig 1:**
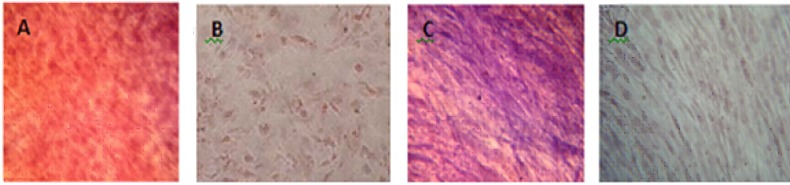
Osteogenic differentiation of Mesenchymal Stem cells (×40). A: Positive reaction in osteoblastic differentiated cells with alizarin red staining; B: Undifferentiated cells; C: Increased alkaline phosphatase activity in osteoblastic differentiated cells; D: Undifferentiated cells

Potential osteogenic differentiation of bone marrow derived MSCs was assayed by alizarin red staining and evaluation of alkaline phosphatase activity. Both alizarin red and alkaline phosphatase activity showed a positive reaction (Figure 1).

***Ex-vivo*** **expansion of CD34+ enriched cells**

**Fig 2:**
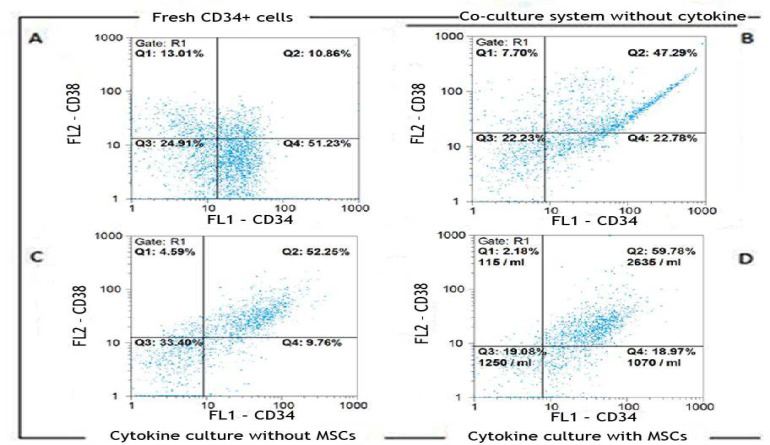
Flow cytometric analysis of the percentage of CD34+ expanded cells in different culture conditions. Flow cytometric analysis of fresh CD34+ enriched cells (A) and expanded cells in the co-culture system without cytokine (B) cytokine culture without MSCs (C) and with MSCs (D) at day 10 of expansion: Specific staining was performed with anti-CD34-FITC and anti-CD38-PE antibodies. FL1: CD34, FL2: CD38; A. CD34: 62.09 %, CD38: 23.87%; B. CD34: 70.07 %, CD38: 54.99%; c. CD34: 62.01 %, CD38: 56.84%; D. CD34: 78.75 %, CD38: 61.96%.

The purity of separated CD34^ +^ cells determi-ned by flow cytometry analysis, was 88% ± 12. Moreover, 31.45% ± 7 of them were positive for CD38 marker. Lineage specific CD markers expression was low (n=3) (Figure 2).

*Ex-vivo* expansion of human cord blood enriched CD34+ cells in serum-free medium supplemented with SCF, TPO and FLT3L was evaluated either with or without feeder layer using flow cytometry analysis. Figures 2B, 2C and 2D show the percentage of CD34+ and CD38+ cells in cells expanded in a co-culture system without cytokine, cytokine cultures without MSCs and with MSCs after 10 days, respectively.

**Fig 3:**
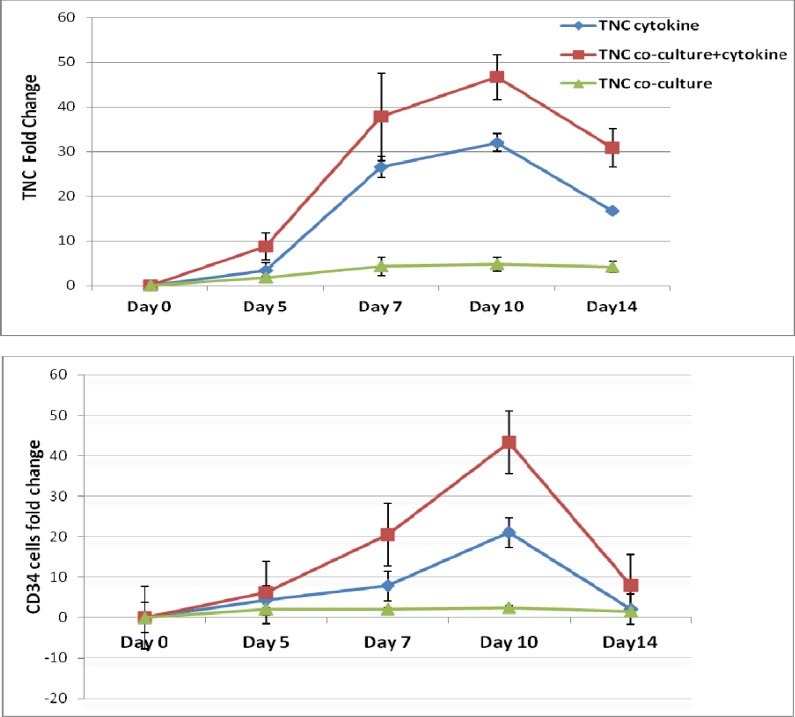
Fold change of TNC & CD34+ cells in different culture conditions. Upper graph: Mean fold change of TNC (Total Nucleated Cell), Lower graph: Mean fold change of CD34+ cells in cytokine culture and co-culture with MSCs supplemented with/ without cytokine

**Fig 4:**
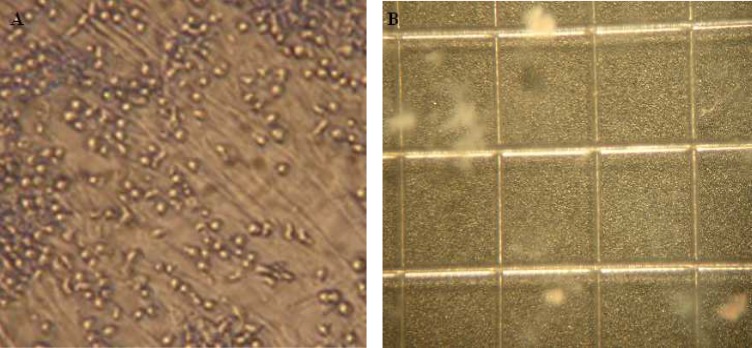
Co-culture of hematopoietic stem cells with MSCs & CFU-assay. A: Co-culture of HSCs with MSCs after 10 days expansion in serum free media supplemented with cytokine cocktail. B: CFU assay of expanded HSCs at day 10 of culture

Figures 3A and 3B show the fold increase in total number of cells (TNC) and number of CD34^+^ cells during 14-day liquid cytokine culture in the presence and absence of human mesenchymal stromal cells. Maximum expansion was observed at the 10^th^ day of culture in all conditions. The mean fold change of TNC was 32 ± 2 in cytokine culture without MSCs feeder layer and 46.6 ± 5 in the co-culture system with cytokines. The mean fold change of CD34^+^ cells was 20.5 ± 7.8 in cytokines supplemented culture and 43.25 ± 8 in the co-culture system with cytokines (n=6) (Figure 4). However, in co-culture system without cytokine, TNC and CD34+ cell numbers were increased up to 4.88 ± 1.5 and 2 folds respectively. 

Expansion of CD34 + selected cord blood cells at 14^th^ day of both cytokine culture and co-culture with cytokines was decreased and a relative differentiation was also observed.

The mean fold change of TNC and CD34+ cells at 10^th^ day in the co-culture system with cytokines was significantly higher than the cytokine culture without MSCs feeder layer and co-culture system without cytokines (n=6, p=0.023). However, in the co-culture system without cytokine, TNC and CD34 + cell numbers increased up to 2 folds and cell viability remained 90% after 14 days.

**Colony-forming cell assays**

**Fig 5:**
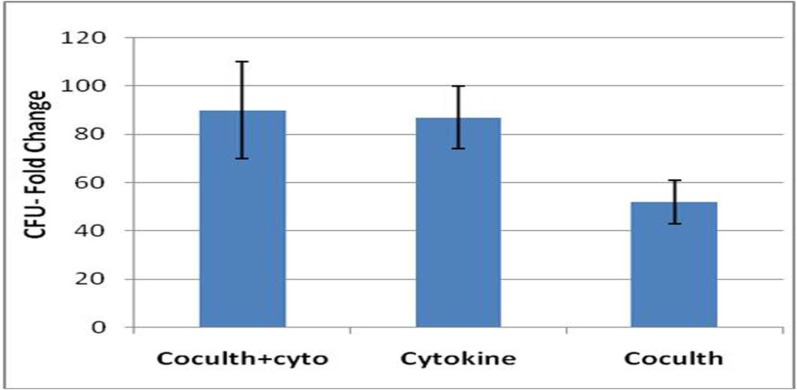
CFU assay of expanded HSCs at day 10 of three culture conditions

The highest CFU fold change was also observed in cytokine culture with MSCs at 10^th^ day (90 ± 24). The CFUs increase in cytokine culture without MSCs at 10^th^ day was 87 ± 13, and in co-culture with MSC was 52 ± 9. So the mean fold change of CFU at 10^th^ day in both cytokine cultures with and without MSCs feeder layer were significantly higher than the co-culture system without cytokines (n=3, p=0.037), but there was no significant differences between the two cytokine cultures with and without MSCs feeder layer (Figures 4, 5).

**Cell Cycle Distribution Analysis**

The distribution of expanded cells in percentage was 56.5 ± 1 at G0/G1, 9.46 ± 1.6 at G2/M and 25.61 ± 1.5 at S phase of cell cycle in co-culture without cytokine. In co-culture with cytokine 41.32 ± 2.3 percent of expanded cells were at G0/G1, 13.07 ± 0.7 percent were at G2/M and 26.69 ± 1 were at S phase of cell cycle and in cytokine culture, distribution in percentage of expanded cells was 30.09 ± 7.9 at G0/G1, 8.29 ± 0.5 at G2/M and 23.65 ± 4.6 S at phase of cell cycle. In every experiment there were some apoptotic cells wherein the percentages were not included. 

**Fig 6:**
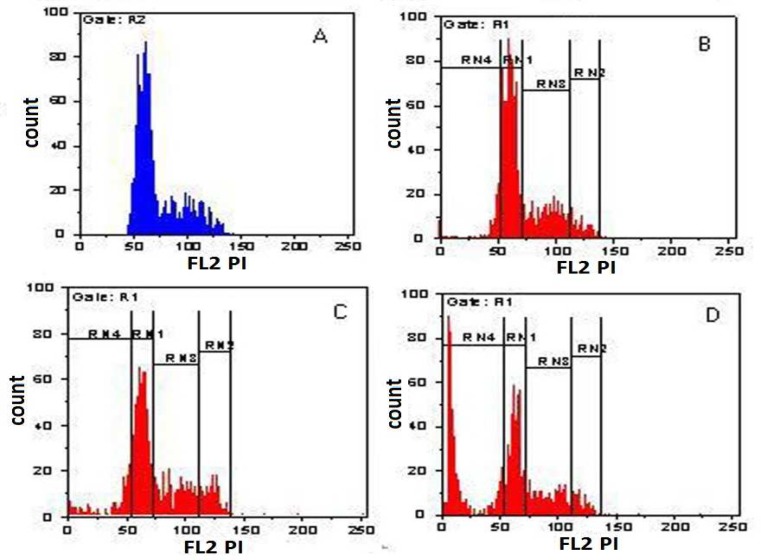
cell cycle distribution analysis of expanded cells. Percentages of cells in A : Control, B: Co-culture without cytokine, C: Co-culture with cytokine, D: Cytokine culture. (RN1) represents G0/G1 phase, (RN2) G2/M phase, (RN3) S phase and (RN4) late apoptosis phase. Gate: R1 counts FL2 PI..

Our results showed that the population of cells in Go/G1 phase in two co-culture condition was significantly higher than cytokine culture and in co-culture without cytokine was also higher than co-culture with cytokine (p=0.041). The population of cells in G2/M phase in co-culture with cytokine was higher than other conditions (p=0.034) But there were no significant differences between the population of cells in S phase (Figure 6).

**Apoptosis analysis**

**Fig 7:**
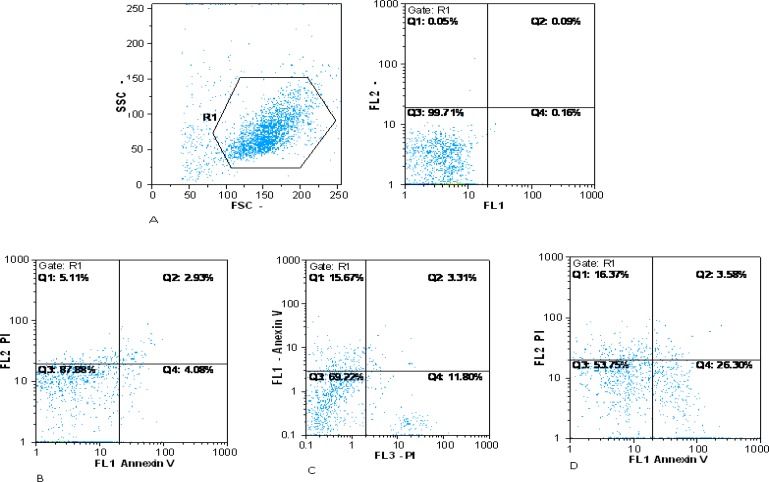
Flow cytometric analysis of apoptosis in different culture conditions. Flow cytometric analysis for apoptosis at day 10 in three culture conditions by PI Staining and Annexin V. A: Negative control; B: Co-culture without cytokine; C: Co-culture with cytokine; D: Cytokine

**Fig 8:**
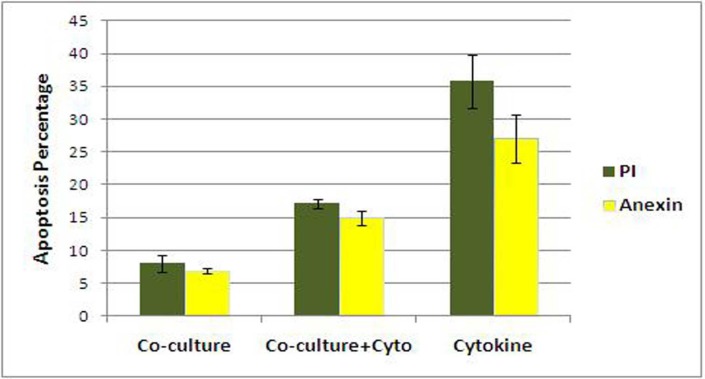
Analysis of apoptosis in different culture conditions. Apoptosis analysis at day 10 of three culture conditions (P<0.005) with PI Staining (late apoptosis) and Annexin V (early apoptosis).

Apoptosis analysis was performed by Annexin V and PI staining on expanded cells cultured in all three culture conditions at day 10^th^ of culture **. **The percentage of apoptotic cells was 8 ± 1.2% PI^+^, 6.86 ± 0.3% Annexin^+^ in co-culture without cytokine, 17.18 ± 0.7% PI^+^, 14.94 ± 1% Annexin^+^ in co-culture with cytokine and 35.77 ± 4.07% PI^+^, 27.03 ± 3.6% Annexin^+^ in cytokine culture. Our results showed that the apoptosis rate in cytokine culture was significantly higher than the two co-cultures condition (P<0.005). Apoptosis rate in co-culture with cytokine was higher than co-culture without cytokine (P<0.005) (Figures 7, 8).

## Discussion

*Ex-vivo* expansion of UCB-HSCs at different combinations of recombinant stimulatory cytokines is one way to increase the number of CD34+ cells and the kinetics of engraftment of UCB-HSCs (9). However, it has been clearly shown that cord blood CD34+ cells with a high number of cell divisions in short-term cultures are associated with an increase in the percentage of apoptotic CD34+ cells (10). Another way is expansion of HSCs in co-culture with mesenchymal stem cells that produce a more “natural” haematopoietic microenvironment for proliferation, self renewal and differentiation of HSCs (11). Prevention of apoptosis is an important strategy to improve stem cell engraftment in preclinical and clinical settings (12). In this study, the effects of cytokines (SCF, Flt3-L and TPO) and mesenchymal stromal layer on cell cycle distribution, apoptosis and proliferation of CB-HSCs CD34+ cells during expansion  were evaluated.

Maximum expansion was observed at the 10^th^ day of culture in the two cytokine supplemented cultures. Moreno et al. (2010) also reported that UCB derived CD34+ cells showed significant proliferation after 14 days in cytokine culture and maximum expansion was observed at the 7^th^ day of culture (13).

The mean fold changes of CD34+ cells at the 10^th^ day of expansion in the two cytokine cultures with and without MSCs were higher than co-culture system without cytokine.  Jang et al. (2006) also showed that the proliferation capacity of HSCs in recombinant cytokine culture with UCB-MSCs feeder layer was higher than the cytokine culture without MSCs, and also in the co-culture system without cytokine CD34+ cell numbers increased up to 3 fold (14). However, in comparison to cytokine cultures, lineage differen-tiation rate was low in the co-culture system without cytokine (data not shown).

Da Silva et al. (2005) cultured CB CD34+ enriched cells in serum-free medium supplemented with SCF, bFGF, LIF and Flt-3, in the presence or absence of stromal layer. They observed that the stromal layers support the process of expansion without more exhaustion of primitive stem cells and differentiation induction (15).

MSCs have been demonstrated to serve as a feeder layer and to maintain HSCs in an undifferentiated state (16). The rate of expansion in culture conditions containing cytokine decreased at 14^th^ day of expansion, but in the co-culture system without cytokine 4 fold expansion was observed constantly during 14 days. Song et al. (2010) claimed that co-cultures of hematopoietic cells with bone marrow derived mesenchymal stem cells without added cytokines, were able to preserve repopulating HSCs for several weeks (17). HSC differentiation during expansion increases HSC senescence and cell death process (18), but direct contact between stem cells and cellular microenvironment has been shown to play an essential role in self-renewal versus differentiation (19).

In this study, we observed that the expansion of HSCs in the cytokine supplemented culture compared to the co-culture system without cytokine have the highest apoptosis rate. Goldenberg et al. (2008) reported that SCF under serum-free conditions induces apoptosis measured by annexin V-PI cell staining, in spite of increasing CD34+ proliferation (18).

Wei et al. (2009) claimed that when K562 cells were co-cultured with normal MSCs, anti-apoptotic gene expression was up regulated and apoptosis rate was decreased (19). Our results showed that using MSCs as a feeder layer for cord blood hematopoietic stem cells caused less apoptosis rate in expanded cells and maintained cells in G0/G1 phase.
